# Association between air pollution and mammographic breast density in the Breast Cancer Surveilance Consortium

**DOI:** 10.1186/s13058-017-0828-3

**Published:** 2017-04-06

**Authors:** Lusine Yaghjyan, Robert Arao, Cole Brokamp, Ellen S. O’Meara, Brian L. Sprague, Gabriela Ghita, Patrick Ryan

**Affiliations:** 1 Department of Epidemiology, College of Public Health and Health Professions and College of Medicine, University of Florida, 2004 Mowry Rd, Gainesville, FL 32610 USA; 2 Group Health Research Institute, 1730 Minor Ave, Seattle, WA 98101 USA; 3 Department of Environmental Health, University of Cincinnati, 3223 Eden Ave, Cincinnati, OH 45267 USA; 4 Department of Surgery, University of Vermont, 111 Colchester Avenue, Burlington, VT 05401 USA; 5 Department of Biostatistics, College of Public Health and Health Professions and College of Medicine, University of Florida, 2004 Mowry Rd, Gainesville, FL 32610 USA; 6 Division of Biostatistics and Epidemiology, Cincinnati Children’s Hospital Medical Center, 3333 Burnet Ave, Cincinnati, OH 45229 USA

**Keywords:** Breast density, Air pollution, Particulate matter, Geographic disparities

## Abstract

**Background:**

Mammographic breast density is a well-established strong risk factor for breast cancer. The environmental contributors to geographic variation in breast density in urban and rural areas are poorly understood. We examined the association between breast density and exposure to ambient air pollutants (particulate matter <2.5 μm in diameter (PM_2.5_) and ozone (O_3_)) in a large population-based screening registry.

**Methods:**

Participants included women undergoing mammography screening at imaging facilities within the Breast Cancer Surveillance Consortium (2001–2009). We included women aged ≥40 years with known residential zip codes before the index mammogram (*n* = 279,967). Breast density was assessed using the American College of Radiology’s Breast Imaging-Reporting and Data System (BI-RADS) four-category breast density classification. PM_2.5_ and O_3_ estimates for grids across the USA (2001–2008) were obtained from the US Environmental Protection Agency Hierarchical Bayesian Model (HBM). For the majority of women (94%), these estimates were available for the year preceding the mammogram date. Association between exposure to air pollutants and density was estimated using polytomous logistic regression, adjusting for potential confounders.

**Results:**

Women with extremely dense breasts had higher mean PM_2.5_ and lower O_3_ exposures than women with fatty breasts (8.97 vs. 8.66 ug/m^3^ and 33.70 vs. 35.82 parts per billion (ppb), respectively). In regression analysis, women with heterogeneously dense vs. scattered fibroglandular breasts were more likely to have higher exposure to PM_2.5_ (fourth vs. first quartile odds ratio (OR) = 1.19, 95% confidence interval (CI) 1.16 − 1.23). Women with extremely dense vs. scattered fibroglandular breasts were less likely to have higher levels of ozone exposure (fourth vs. first quartile OR = 0.80, 95% CI 0.73–0.87).

**Conclusion:**

Exposure to PM_2.5_ and O_3_ may in part explain geographical variation in mammographic density. Further studies are warranted to determine the causal nature of these associations.

## Background

Mammographic breast density is a well-established independent risk factor for breast cancer [1]. Previous studies suggest there are differences in breast density among women living in urban and rural areas [2, 3]. A recent report suggested that women in urban areas may have higher breast density as compared to those living in the rural environment, but these results may have been confounded by body mass index (BMI); these differences were more prominent among women age 45–54 years [3]. The etiology of higher density in urban areas is unclear and whether environmental exposures could contribute to these patterns is unknown. Environmental factors such as air pollution may contribute to geographic variation in breast density because urban and rural areas have distinct air pollution patterns [4–7] and some air pollutants are known to have endocrine-disrupting properties [8–13].

The evidence on association between air pollution and breast cancer is limited. In some previous studies there have been reports of positive associations between risk of breast cancer and nitrogen dioxide (NO_2_), fine particles <2.5 μm in diameter (PM_2.5_), polycyclic aromatic hydrocarbons (PAHs), carbon monoxide, sulfur dioxide, and volatile organic compounds [14–17], and a positive association between PM_2.5_ and risk of death from breast cancer [18]. In other studies there has been no association between breast cancer and PM_2.5_, total suspended particles, ozone, or particles 2.5–10 μm in diameter (PM_10_) [19, 20]. In analyses stratified by tumor subtype, some of the air pollutants were associated with estrogen-receptor-positive and progesterone-receptor-positive (ER+ and PR+) tumors; other constituents have been associated with receptor-negative subtypes only. Positive associations have been identified between ER+/PR+ tumors and ambient levels of NO_2_, acrylamide, benzidine, carbon tetrachloride, ethylidene dichloride, and vinyl chloride, and between ER-negative/PR-negative (ER-/PR-) subtypes and ambient levels of benzene, cadmium, and inorganic arsenic [19, 21, 22].

Whether air pollution could contribute to high breast density is unknown. In the only study in which the association between breast density and nitrogen oxides or NO_2_ was investigated, there was a borderline inverse association between air pollution and the risk of mixed/dense breast density patterns [23]. To add to the limited knowledge on the association between air pollution and mammographic breast density, we examined the association between breast density and PM_2.5_ and ozone (O_3_) in a large consortium of population-based mammography registries. We further examined the interactions of PM_2.5_ and ozone with menopausal status and use of postmenopausal hormones, family history of breast cancer, and BMI.

## Methods

### Study population

Women in this study were selected from participants in the Breast Cancer Surveillance Consortium (BCSC) (http://www.bcsc-research.org/), which is funded by the National Cancer Institute, and is a population-based prospective cohort of women undergoing mammographic screening at facilities affiliated with a network of breast imaging registries throughout the USA. Five BCSC registries were included in this analysis: New Hampshire Mammography Network, Vermont Breast Cancer Surveillance System, New Mexico Mammography Project, San Francisco Mammography Registry, and Group Health Cooperative in western Washington State.

The BCSC has been described in detail elsewhere [24–26]. Briefly, each BCSC registry collects clinical information on diagnostic and screening mammography examinations in their defined catchment areas. Information on demographics and risk factors is collected by questionnaire administered at each mammographic examination. Information on malignant and benign diagnoses is retrieved via linkage of the registry data with the state tumor registry or regional Surveillance Epidemiology and End Results programs and with pathology databases.

**Fig. 1:**
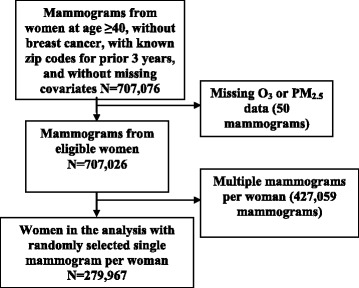
Participant selection diagram. *O*_*3*_ ozone, *PM*_*2.*5_ particulate matter <2.5 μm in diameter

Women were included in our analysis if they had a screening mammogram between 2001 and 2009 with data available on breast density, were age ≥40 years, had no previous history of breast cancer, had a known residential zip code for at least 3 years prior to the index mammogram date, and non-missing data on the covariates used in the analysis. For women with multiple mammograms, we randomly selected one mammogram for which residential zip code data were available that satisfied the 3-year residential zip code requirement. The final study population included 279,967 women (Fig. 1).

Each mammography registry and the Statistical Coordinating Center (SCC) have received Institutional Review Board approval for either active or passive consenting processes or a waiver of consent to enroll participants, link data, and perform analytic studies. All procedures comply with the Health Insurance Portability and Accountability Act, and all registries and the SCC have received a Federal Certificate of Confidentiality and other protection for the identities of women, physicians, and facilities studied by this research.

### Mammographic breast density

Categorical mammographic breast density was defined using the American College of Radiology’s Breast Imaging-Reporting and Data System (BI-RADS) breast density classification recorded by the clinician on the woman’s screening mammogram (category 1 (BI-RADS I) - predominantly fat, category 2 (BI-RADS II) - fat with some fibroglandular tissue (reference), category 3 (BI-RADS III) - heterogeneously dense, and category 4 (BI-RADS IV) - extremely dense). In the fourth edition of the BI-RADS manual (2003) [27], the percentage of glandular material was added to the density definition as follows: <25% glandular (category 1), 25–50% glandular (category 2), 51–75% glandular (category 3), and >75% glandular (category 4). As our study included mammograms performed between 2001 and 2009, density definitions from both the third and fourth editions were used [28].

### Air pollution exposure assessment

Exposure data for PM_2.5_ and O_3_ estimates for grids across the USA from 2001 to 2008 were obtained from the US Environmental Protection Agency Hierarchical Bayesian Model (HBM), which combines monitoring data with numerical output from the Community Multi-scale Air Quality model [29]. For both pollutants, the smallest grids available for each year were used (2001–2006: 36 km; 2007–2008: 12 km). Daily 24-hour mean concentrations of PM_2.5_ and 8-hour maximum concentrations of O_3_ were used to calculate annual concentrations for each grid. Yearly mean exposures of PM_2.5_ and O_3_ for each subject were calculated using the inverse distance-weighted method based on their zip code centroid and HBM grid centroids. For each zip code centroid, the distance to the centroids of the four closest HBM grids were calculated based on the “Haversine” great circle distance using the latitude and longitude coordinates. The four closest distances were assigned a weight:

$$ {w}_i=\frac{\frac{1}{d_i^2}}{{\displaystyle {\sum}_{i=1}^4}\frac{1}{d_i^2}} $$

where d_i_ is the distance between the zip code centroid and each of the four closest HBM grid centroids. The yearly mean exposure for the zip code was then calculated as the weighted average:

$$ {\displaystyle \sum_{i=4}^4}{w}_i\ast {P}_i $$

where P_i_ is the pollutant concentration.

PM_2.5_ and O_3_ exposures for the year preceding the mammogram were retrieved for analysis. Data were unavailable for the preceding year in 6% of the study sample, and thus the exposure estimates were retrieved for the year of the mammogram. The PM_2.5_ and O_3_ exposures were modeled as continuous variables and as quartiles based on their distribution in the study population (<7.91, 7.91 to <8.81, 8.81 to <9.86, and ≥9.86 ug/m^3^ for PM_2.5_; <29.73, 29.73 to <36.05, 36.05 to <37.92, and ≥37.92 parts per billion (ppb) for O_3_). In a secondary analysis, we also used the exposure data from the year of the mammogram in all women to examine the association between exposure and breast density.

### Covariates

Information on covariates was available from the date of the index mammogram. We included the following potential confounders in the models: age, race/ethnicity, BMI, study site, menopausal status/use of hormone replacement therapy, age at menarche, parity and age at first birth, history of breast biopsy, family history of breast cancer, and median household income for the zip code.

### Statistical analysis

We used multivariate polytomous regression to examine the associations between categorical data on breast density and exposure to PM_2.5_ and O_3_. In these models, women with predominantly fat, heterogeneously dense, or extremely dense breasts were compared to women with scattered fibroglandular density (BI-RADS II). Each air pollution variable was modeled as a continuous variable and as quartiles based on the distribution in the study population. The lowest exposure category was used as the reference in all analyses. A two-sided test for trend was performed, modeling each air pollutant as an ordinal variable and using the median air pollutant level in each category. The risk estimates in all analyses were adjusted for age (40–49, 50–59, 60–69, 70–79, or 80–89 years), race/ethnicity (white, black, Asian/Pacific Islander, American Indian, Hispanic, or mixed/other), BMI (≤18.4, 18.5–24.9, 25.0–29.9, 30.0–34.9, 35.0–39.9, or ≥40 kg/m^2^), study site, menopausal status/use of hormone replacement therapy (premenopausal, perimenopausal, postmenopausal not using hormone replacement therapy, postmenopausal currently using hormone replacement therapy), age at menarche (≤12, 13, 14, or ≥15 years), parity and age at first birth (nulliparous, parous with age at first birth ≤29 years, or parous with age at first birth ≥30 years), history of breast biopsy (yes or no), first-degree family history of breast cancer (yes or no), and median household income for the zip code (≤US$46,075, US$46,076–54,093, US$54,094–66,322, or ≥ US$66,323).

We next examined the two-way interactions between each of the exposures and BMI, family history of breast cancer, history of breast biopsy, or menopausal status/postmenopausal hormone use. To test these interactions, we implemented different approaches by using both continuous exposure variables and the respective medians within each of the exposure categories, to model the interaction term. The results were similar with both approaches. BMI was modeled as a binary variable (<30 kg/m^2^ vs. ≥30 kg/m^2^) and both family history of breast cancer and menopausal status/hormone replacement therapy use were modeled as nominal data. Statistical significance in all analyses was assessed at the 0.05 level. Finally, for variables that had significant interactions, we examined the association between exposure and breast density separately across the variable strata. The analyses were performed using SAS software (version 9.3, SAS Institute, Cary, NC, USA).

## Results

Characteristics of the 279,967 women included in this study are presented in Table 1. The mean age of the participants was 57 years (range 40–102) and the majority of the women were postmenopausal (70.9%). The distribution of BI-RADS density categories was 10.8%, 41.7%, 39.4%, and 8.2% for BI-RADS I, II, II, and IV, respectively, and was consistent with previously reported distributions in women of screening age [24, 30–33]. Among all women, 60.3% were living in urban areas and 39.7% in rural areas. Mean concentrations of PM_2.5_ were higher in women with higher breast density (8.77, 8.88, 9.24, and 9.34 ug/m^3^ for BI-RADS I, II, III, and IV respectively) and concentrations of O_3_ were lower in women with higher density (35.95, 35.22, 34.04, and 33.72 ppb for BI-RADS I, II, III, and IV respectively).

**Table 1:** Characteristic of the study population (*n* = 279,967), by breast density category (number [percentage])

Characteristics	Almost entirely fat (BI-RADS I) *n* = 30,249	Scattered fibroglandular densities (BI-RADS II) *n* = 116,666	Heterogeneously dense (BI-RADS III) *n* = 110,181	Extremely dense (BI-RADS IV) *n* = 22,871
Age at mammogram, years				
40–49	4526 (15.0)	27,195 (23.3)	38,172 (34.6)	11,261 (49.2)
50–59	9548 (31.6)	37,768 (32.4)	37,004 (33.6)	7432 (32.5)
60–69	8749 (28.9)	28,004 (24.0)	19,960 (18.1)	2536 (11.1)
70–79	5388 (17.8)	16,712 (14.3)	10,331 (9.4)	1069 (4.7)
80–89	2038 (6.7)	6987 (6.0)	4714 (4.3)	573 (2.5)
Race/ethnicity				
White	25,879 (85.6)	101,078 (86.6)	92,698 (84.1)	18,170 (79.4)
Black	703 (2.3)	2031 (1.7)	1873 (1.7)	352 (1.5)
Asian/Pacific Islander	842 (2.8)	4085 (3.5)	7416 (6.7)	2662 (11.6)
American Indian	117 (0.4)	442 (0.4)	373 (0.3)	45 (0.2)
Hispanic	2056 (6.8)	6248 (5.4)	5122 (4.6)	1115 (4.9)
Mixed/other	652 (2.2)	2782 (2.4)	2699 (2.4)	527 (2.3)
Body mass index, kg/m^2^				
≤18.4	142 (0.5)	956 (0.8)	1808 (1.6)	1198 (5.2)
18.5–24.9	5383 (17.8)	36,175 (31.0)	53,604 (48.7)	15,974 (69.8)
25.0–29.9	8846 (29.2)	38,576 (33.1)	33,316 (30.2)	4283 (18.7)
30.0–34.9	7548 (25.0)	23,267 (19.9)	13,803 (12.5)	1038 (4.5)
35.0–39.9	4361 (14.4)	10,501 (9.0)	5028 (4.6)	273 (1.2)
≥40	3969 (13.1)	7191 (6.2)	2622 (2.4)	105 (0.5)
Age at menarche, years				
≤12	14,400 (47.6)	51,281 (44.0)	44,093 (40.0)	7989 (34.9)
13	9211 (30.5)	37,191 (31.9)	35,622 (32.3)	7516 (32.9)
14	3626 (12.0)	15,203 (13.0)	15,816 (14.4)	3682 (16.1)
≥15	3012 (10.0)	12,991 (11.1)	14,650 (13.3)	3684 (16.1)
Parity/age at first child				
Nulliparous	4019 (13.3)	14,264 (12.2)	18,085 (16.4)	5727 (25.0)
Parous/≤29 years	23,317 (77.1)	88,215 (75.6)	74,309 (67.4)	11,896 (52.0)
Parous/≥30 years	2913 (9.6)	14,187 (12.2)	17,787 (16.1)	5248 (22.9)
Menopausal status/HRT				
Premenopausal	3776 (12.5)	24,161 (20.7)	35,502 (32.2)	10,855 (47.5)
Postmenopausal/current HRT use	3064 (10.1)	14,627 (12.5)	15,995 (14.5)	2840 (12.4)
Postmenopausal/no current HRT use	22,908 (75.7)	75,358 (64.6)	55,548 (50.4)	8378 (36.6)
Perimenopausal	501 (1.7)	2520 (2.2)	3136 (2.8)	798 (3.5)
Family history of breast cancer				
Yes	5099 (16.9)	19,787 (17.0)	18,946 (17.2)	3860 (16.9)
No	25,150 (83.1)	96,879 (83.0)	91,235 (82.8)	19,011 (83.1)
History of breast biopsy				
Yes	5250 (17.4)	23,387 (20.0)	26,687 (24.2)	6255 (27.3)
No	24,999 (82.6)	93,279 (80.0)	83,494 (75.8)	16,616 (72.7)
Residential area				
Urban	17,673 (59.1)	63,393 (54.6)	72,210 (65.6)	14,933 (65.4)
Rural	12,244 (40.9)	52,816 (45.4)	37,794 (34.4)	7916 (34.6)
Median household income				
≤US$46,075	8811 (29.1)	33,011 (28.3)	24,474 (22.2)	4591 (20.1)
US$46,076–54,093	7871 (26.0)	31,185 (26.7)	25,607 (23.2)	5324 (23.3)
US$54,094–66,322	7051 (23.3)	28,168 (24.1)	30,237 (27.4)	5897 (25.8)
≥US$66,323	6516 (21.5)	24,302 (20.8)	29,863 (27.1)	7059 (30.9)
PM_2.5_ (ug/m^3^)				
Mean (SD)	8.77	8.88	9.24	9.34
Range	2.86–19.63	2.39–19.83	2.19–23.05	2.54–18.57
Ozone (ppb)				
Mean (SD)	35.95	35.22	34.04	33.72
Range	24.54–53.65	22.30–57.39	24.54–54.22	25.46–50.81

*Abbreviations: BI-RADS* American College of Radiology’s Breast Imaging-Reporting and Data System; *HRT* hormone replacement therapy, *PM*_*2.5*_ particulate matter <2.5 μm in diameter

In multivariate regression analysis, women with heterogeneously dense vs. scattered fibroglandular breasts were more likely to have been exposed to higher concentrations of PM_2.5_ (fourth vs. first quartile odds ratio (OR) = 1.19, 95% confidence interval (CI) 1.16–1.23; third vs. first quartile OR = 1.19, 95% CI 1.16–1.22) and women with fatty breasts were less likely to have been exposed to higher levels of PM _2.5_ (fourth vs. first quartile OR = 0.88, 95% CI 0.85–0.92; third vs. first quartile OR = 0.85, 95% CI 0.81–0.88) (Table 2). A one-unit increase in PM_2.5_ concentration was associated with 4% increased chance of having heterogeneously dense breasts and 2% lower chance of having fatty breasts vs. scattered fibroglandular breasts. Women with extremely dense breasts vs. scattered fibroglandular breasts were less likely to have been exposed to higher levels of O_3_ (fourth vs. first quartile OR = 0.80, 95% CI 0.73–0.87) and women with fatty breasts were more likely to have been exposed to higher O_3_ concentrations (fourth vs. first quartile OR = 1.12, 95% CI 1.04–1.20) (Table 2). A one-unit increase in O_3_ concentration was associated with 3% lower chance of having extremely dense breasts and 2% higher chance of having fatty breasts, vs. scattered fibroglandular breasts.

**Table 2:** Associations of PM_2.5_ and O_3_ with breast density

Exposure	BI-RADS I OR (95% CI)	BI-RADS III OR (95% CI)	BI-RADS IV OR (95% CI)
PM_2.5_ quartile (median, ug/m^3^)^a^			
1^st^ (7.18)	Referent	Referent	Referent
2^nd^ (8.44)	0.95 (0.92–0.98)	1.08 (1.05–1.11)	0.90 (0.86–0.94)
3^rd^ (9.27)	0.85 (0.81–0.88)	1.19 (1.16–1.22)	1.00 (0.95–1.05)
4^th^ (10.68)	0.88 (0.85–0.92)	1.19 (1.16–1.23)	0.97 (0.92–1.02)
*P* for trend	<0.0001	<0.0001	<0.0001
Continuous PM_2.5_	0.98 (0.97–0.99)	1.04 (1.03–1.04)	1.00 (0.99–1.01)
O_3_ quartile (median, ppb)^a^			
1^st^ (28.48)	Referent	Referent	Referent
2^nd^ (32.94)	0.86 (0.81–0.90)	1.13 (1.10–1.16)	1.08 (1.03–1.14)
3^rd^ (37.03)	0.85 (0.79–0.91)	1.11 (1.07–1.16)	0.99 (0.92–1.07)
4^th^ (39.05)	1.12 (1.04–1.20)	0.98 (0.94–1.03)	0.80 (0.73–0.87)
*P* for trend	<0.0001	<0.0001	<0.0001
Continuous O_3_	1.02 (1.01–1.03)	1.00 (1.00–1.01)	0.97 (0.96–0.98)

For breast density, American College of Radiology’s Breast Imaging-Reporting and Data System (BI-RADS) II (scattered fibroglandular densities) is the reference category; risk estimates are adjusted for age at mammogram, body mass index at mammogram, race, study site, age at menarche, parity and age at first birth, menopausal status/hormone use, family history of breast cancer, history of breast biopsy, and median household income for the zip code. ^a^Quartiles defined as <7.91, 7.91 to <8.81, 8.81 to <9.86, and ≥9.86 ug/m^3^ for particulate matter <2.5 μm in diameter (PM_2.5_) and <29.73, 29.73 to <36.05, 36.05 to <37.92 and ≥37.92 ppb for ozone (O_3_)

*CI* confidence interval, *HRT* hormone replacement therapy, *OR* odds ratio. Note: BI-RADS II is the reference group

We found significant interactions of both exposure variables with menopausal status/hormone replacement therapy use (*p* for interaction 0.02 and <0.0001 for PM_2.5_ and O_3_, respectively) and BMI *p* for interaction <0.01 and <0.0001 for PM_2.5_ and O_3_, respectively) (Table 3). Despite the statistical significance of the interactions, there did not appear to be substantial, clinically relevant differences in the direction or magnitude of the associations across the strata of the effect modifier. The findings were similar in a secondary analysis using the exposure data from the year of the mammograms (data not shown).

**Table 3:** Associations of PM_2.5_ and O_3_ with breast density, stratified by body mass index, family history of breast cancer, and menopausal status/postmenopausal hormone use

	Pollutant quartile^a^	PM_2.5_ (ug/m^3^)			O_3_ (ppb)		
Analytical strata		BI-RADS I OR (95% CI)	BI-RADS III OR (95% CI)	BI-RADS IV OR (95% CI)	BI-RADS I OR (95% CI)	BI-RADS III OR (95% CI)	BI-RADS IV OR (95% CI)
BMI <30^b^							
	1^st^	Referent	Referent	Referent	Referent	Referent	Referent
	2^nd^	1.01 (0.96–1.06)	1.06 (1.03–1.09)	0.88 (0.84–0.92)	0.97 (0.90–1.04)	1.15 (1.11–1.20)	1.10 (1.04–1.16)
	3^rd^	0.93 (0.88–0.98)	1.16 (1.13–1.20)	0.98 (0.94–1.03)	0.87 (0.79–0.96)	1.14 (1.09–1.20)	1.02 (0.94–1.10)
	4^th^	0.95 (0.90–1.01)	1.16 (1.12–1.20)	0.94 (0.89–0.99)	1.12 (1.01–1.25)	1.02 (0.96–1.07)	0.85 (0.78–0.93)
BMI ≥30^b^							
	1^st^	Referent	Referent	Referent	Referent	Referent	Referent
	2^nd^	0.91 (0.86–0.95)	1.16 (1.10–1.22)	1.20 (1.02–1.41)	0.78 (0.73–0.84)	1.07 (1.01–1.12)	1.03 (0.88–1.21)
	3^rd^	0.79 (0.75–0.84)	1.27 (1.20–1.33)	1.05 (0.89–1.24)	0.84 (0.76–0.93)	1.06 (0.97–1.15)	0.94 (0.73–1.20)
	4^th^	0.85 (0.81–0.91)	1.27 (1.20–1.34)	1.09 (0.91–1.30)	1.13 (1.02–1.25)	0.93 (0.85–1.02)	0.62 (0.46–0.82)
Premenopausal^c^							
	1^st^	Referent	Referent	Referent	Referent	Referent	Referent
	2^nd^	0.96 (0.87–1.06)	1.06 (1.01–1.12)	0.92 (0.85–0.99)	0.85 (0.72–1.00)	1.17 (1.09–1.26)	1.16 (1.06–1.26)
	3^rd^	0.82 (0.74–0.91)	1.25 (1.19–1.32)	1.06 (0.98–1.15)	0.82 (0.67–1.00)	1.25 (1.14–1.37)	1.16 (1.03–1.32)
	4^th^	0.86 (0.77–0.96)	1.20 (1.13–1.26)	0.97 (0.89–1.05)	1.04 (0.84–1.28)	1.21 (1.10–1.33)	0.99 (0.86–1.13)
Postmenopausal/no HRT^c^							
	1^st^	Referent	Referent	Referent	Referent	Referent	Referent
	2^nd^	0.94 (0.91–0.98)	1.08 (1.05–1.12)	0.90 (0.84–0.97)	0.85 (0.80–0.90)	1.10 (1.06–1.14)	1.06 (0.99–1.13)
	3^rd^	0.83 (0.79–0.87)	1.15 (1.11–1.19)	0.97 (0.90–1.04)	0.83 (0.77–0.90)	1.05 (0.99–1.11)	0.87 (0.78–0.98)
	4^th^	0.88 (0.84–0.92)	1.21 (1.17–1.25)	1.01 (0.94–1.09)	1.11 (1.02–1.21)	0.88 (0.83–0.94)	0.69 (0.61–0.79)
Postmenopausal/with HRT^c^							
	1^st^	Referent	Referent	Referent	Referent	Referent	Referent
	2^nd^	0.98 (0.87–1.10)	1.14 (1.06–1.22)	0.88 (0.77–1.00)	0.90 (0.77–1.06)	1.21 (1.12–1.30)	1.04 (0.91–1.19)
	3^rd^	0.96 (0.84–1.09)	1.29 (1.20–1.39)	1.08 (0.95–1.24)	0.86 (0.70–1.07)	1.19 (1.06–1.33)	1.08 (0.87–1.33)
	4^th^	1.00 (0.87–1.14)	1.15 (1.06–1.24)	0.96 (0.83–1.10)	1.02 (0.81–1.30)	1.03 (0.90–1.18)	0.80 (0.62–1.03)
Perimenopausal^c^							
	1^st^	Referent	Referent	Referent	Referent	Referent	Referent
	2^nd^	0.97 (0.74–1.28)	0.98 (0.83–1.15)	0.93 (0.72–1.21)	0.83 (0.54–1.27)	0.94 (0.78–1.14)	0.93 (0.70–1.23)
	3^rd^	1.06 (0.79–1.41)	1.16 (0.98–1.37)	1.03 (0.79–1.34)	0.95 (0.54–1.65)	0.89 (0.68–1.17)	0.75 (0.48–1.16)
	4^th^	0.95 (0.69–1.30)	1.34 (1.12–1.60)	1.07 (0.80–1.42)	1.29 (0.72–2.31)	0.76 (0.56–1.03)	0.62 (0.38–1.02)
No Family history of breast cancer^d^							
	1^st^	Referent	Referent	Referent	Referent	Referent	Referent
	2^nd^	0.93 (0.89–0.97)	1.08 (1.05–1.12)	0.90 (0.85–0.94)	0.87 (0.82–0.92)	1.12 (1.09–1.16)	1.10 (1.04–1.16)
	3^rd^	0.83 (0.80–0.87)	1.20 (1.16–1.23)	1.00 (0.95–1.05)	0.85 (0.79–0.92)	1.11 (1.06–1.16)	0.99 (0.91–1.07)
	4^th^	0.87 (0.83–0.91)	1.21 (1.17–1.25)	0.95 (0.90–1.01)	1.11 (1.03–1.21)	0.98 (0.93–1.03)	0.80 (0.73–0.88)
Family history of breast cancer^d^							
	1^st^	Referent	Referent	Referent	Referent	Referent	Referent
	2^nd^	1.05 (0.96–1.14)	1.05 (0.99–1.12)	0.92 (0.82–1.03)	0.79 (0.70–0.90)	1.15 (1.08–1.24)	1.02 (0.90–1.14)
	3^rd^	0.90 (0.82–0.99)	1.17 (1.10–1.24)	0.99 (0.88–1.11)	0.82 (0.69–0.98)	1.15 (1.04–1.28)	1.02 (0.85–1.22)
	4^th^	0.93 (0.84–1.03)	1.12 (1.05–1.20)	1.05 (0.94–1.18)	1.13 (0.94–1.35)	1.01 (0.90–1.13)	0.77 (0.63–0.94)

^a^Quartiles defined as <7.91, 7.91 to <8.81, 8.81 to <9.86, and ≥9.86 ug/m^3^ for particulate matter <2.5 μm in diameter (PM_2.5_) and <29.73, 29.73 to <36.05, 36.05 to <37.92 and ≥37.92 ppb for ozone (O_3_). ^b^Adjusted for age at mammogram, race, study site, age at menarche, parity and age at first birth, menopausal status/hormone use, family history of breast cancer, history of breast biopsy, and median household income for the zip code. ^c^Adjusted for age at mammogram, body mass index (BMI) at mammogram, race, study site, age at menarche, parity and age at first birth, family history of breast cancer, history of breast biopsy, and median household income for the zip code

^d^Adjusted for age at mammogram, BMI at mammogram, race, study site, age at menarche, parity and age at first birth, menopausal status/hormone use, family history of breast cancer, history of breast biopsy, and median household income for the zip code. American College of Radiology’s Breast Imaging-Reporting and Data System (BI-RADS) II (scattered fibroglandular densities) is the reference group. *CI* confidence interval, *OR* odds ratio. *P* values for two-way interactions: PM_2.5_ with BMI *p* < 0.01; PM_2.5_ with menopausal status/hormone replacement therapy (HRT) *p* = 0.02; PM_2.5_ with family history of breast cancer *p* = 0.21; O_3_ with BMI *p* < 0.0001; O_3_ with menopausal status/HRT *p* < 0.0001; O_3_ with family history of breast cancer *p* = 0.56

## Discussion

We examined the associations between PM_2.5_ or O_3_ and mammographic breast density in a large population-based sample of cancer-free women in the Breast Cancer Surveillance Consortium. We found positive associations between breast density and PM_2.5_ and inverse associations between breast density and O_3_.

In a recent study in the Danish Diet, Cancer and Health cohort (1993–1997) the association between mammographic breast density (defined as mixed/dense or fatty breasts) and traffic-related air pollution (modeled as levels of nitrogen oxides (NO_x_) and nitrogen dioxide (NO_2_)) was evaluated among 4769 women [23]. There was a borderline-significant inverse association between long-term exposure and breast density (OR 0.96, 95% CI 0.93–1.01 per 20 μg/m^3^ of NO_x_ and OR 0.89, 95% CI 0.80–0.98 per 10 μg/m^3^ of NO_2_) and no interaction between the exposure and menopause, smoking, or obesity [23]. In contrast, in our analyses we examined the association between breast density and PM_2.5_ or O_3_ levels. Further, we used the BI-RADS density classification in our study with BI-RADS II (scattered fibroglandular breasts) as the reference group, rather than collapsing four density categories into two groups. We also observed significant interactions of both air pollutants with menopausal status and hormone therapy use and BMI. These differences between studies could be potentially explained by the significantly larger sample size in our study, the different classification of density and the approaches used to characterize air pollution.

The chemical composition of PM_2.5_ is diverse and is represented by a variety of compounds, including those with endocrine-disrupting and carcinogenic properties. Specifically, PAHs that represent a relatively small mass percentage in PM_2.5_, are known to have endocrine-disrupting properties and cause adverse effects [8–10]. PAHs also interfere with normal DNA repair processes by forming persistent DNA adducts [34–36]. Among other organic compounds found in PM_2.5_ polychlorinated dibenzodioxins (dioxin), dibenzofurans (PCDF), and biphenyls (PCB) have also been shown to interfere with normal endocrine function [9, 11]. Finally, some of the heavy metals such as cadmium, arsenic and mercury that are found in fine particulate matter also have endocrine-disrupting properties [12, 13]. As mammographic breast density is a reflection of relative amounts of epithelial, stromal and fat tissue and as epithelial proliferation is regulated by a variety of hormonal influences including estrogens and growth factors, these chemical constituents could potentially influence breast density by interfering with normal proliferation, thus increasing the relative amount of fibroglandular tissue in the breast, and subsequently, breast density.

Previous studies have consistently linked higher levels of O_3_ to higher levels of oxidative stress and demonstrated that biological properties of O_3_ can cause many deleterious effects, including cellular death, in distant tissues [37]. Recent studies suggest inhibition of cellular growth in tumor tissue from different organs, including the breast, at the level of 0.3 ppm [38]. It is possible that the effect of O_3_ on breast tissue could result from these inhibitory processes resulting in a lower percentage of fibroglandular structures, and subsequently, lower breast density. However, the causal links, if any, and the underlying biological mechanisms behind the associations of air pollution with breast density need to be elucidated and confirmed.

Previous studies suggest that associations between breast density and risk factors for breast cancer differ by menopausal status, hormone use, family history of breast cancer, and BMI [39–42]. Premenopausal and postmenopausal women differ with respect to the endogenous and exogenous hormonal influences and proliferative activity in the breast tissue and thus menopausal status/hormone use may potentially modify the association between air pollution and breast density. Family history of breast cancer and BMI may reflect the differences in individuals’ genetic factors and xenobiotic metabolism rates. Further, some of the lipophilic xenobiotics, such as PAHs, dioxin, and PCBs may accumulate in adipose tissue, including that in the breast, and as a result, the associations between these chemicals and breast density may differ by BMI. Even though we found statistically significant interactions of both exposures with menopausal status/hormone use and BMI, the observed differences in the risk estimates for PM_2.5_ across the strata were small. The findings suggested a stronger association between O_3_ and breast density in women with BMI ≥30. The patterns of association across categories of menopausal status/hormone use, however, were inconsistent.

Our study utilized an established consortium of population-based mammography registries with information on breast cancer risk factors, demographics, residential history, and breast density. To our knowledge, this is the largest study to date to examine the association between air pollution and breast density and the first study to explore the associations between breast density and PM_2.5_ or O_3_.

Our study has a few limitations. We controlled for known determinants of density in our analysis; however, residual confounding cannot be ruled out. The risk estimates in our study were not adjusted for smoking status. However, the findings on associations between smoking and breast density in previous studies have been inconsistent [41–46]. Further, the additional adjustment for smoking status in the study of air pollution and breast density by Huynh et al. did not change the risk estimates [23]. In our study the risk estimates for PM_2.5_ for BI-RADS IV were not statistically significant. Even though BI-RADS IV represented the smallest density group, the absolute number of women in this category was sufficiently large to detect significant associations. The biological explanation for an association with BI-RADS III density but not BI-RADS IV density is unclear and confirmation of these findings in other populations is warranted.

We used data from Environmental Protection Agency (EPA) air monitoring rather than individual-level exposure data; thus, exposure misclassification cannot be excluded, as our model assumed that the pollutant concentrations were equal throughout each HBM grid. However, this exposure misclassification would likely be non-differential and thus could drive our results towards the null. Using an exposure assessment model with a higher spatial resolution, like land use modeling, was not feasible with only zip code information from the women. Using the community multiscale air quality (CMAQ) model allowed us to assess exposure for locations and days that might be otherwise missing from monitoring stations. Furthermore, the CMAQ model has been shown to be more accurate and precise than interpolation of monitoring data with ordinary kriging regression [29].

## Conclusions

In conclusion, in this large population-based sample of cancer-free women, we found positive associations between and PM_2.5_ and mammographic breast density and inverse associations between ozone and mammographic breast density. Our findings suggest that previously reported geographic variation in breast density could in part be explained by different air pollution patterns in urban and rural areas. Future studies are warranted to determine the causal nature of these associations and to explore whether breast density mediates the ffect of air pollution on breast cancer risk.

## Abbreviations

BCSC: Breast Cancer Surveillance Consortium
BI-RADS: American College of Radiology’s Breast Imaging-Reporting and Data System
BMI: Body mass index
CI: Confidence interval
CMAQ: Community multiscale air quality
ER: Estrogen receptor
HBM: Hierarchical Bayesian model
NO_2_: Nitrogen dioxide
O_3_: Ozone
OR: Odds ratio
PAHs: Polycyclic aromatic hydrocarbons
PM_10_: Particles 2.5–10 μm in diameter
PM_2.5_: Particulate matter <2.5 μm in diameter
PR: Progesterone receptor
ppb: Parts per billion
SCC: Statistical Coordinating Center
